# Direct Growth Inhibitory Effect of Platelet Activating Factor C-16 and Its Structural Analogs on Mycobacteria

**DOI:** 10.3389/fmicb.2018.01903

**Published:** 2018-09-11

**Authors:** Muhammad S. Riaz, Anuvinder Kaur, Suha N. Shwayat, Shahriar Behboudi, Uday Kishore, Ansar A. Pathan

**Affiliations:** ^1^College of Health and Life Sciences, Division of Biosciences, Brunel University London, Uxbridge, United Kingdom; ^2^Department of Biotechnology, Abdul Wali Khan University Mardan, Mardan, Pakistan; ^3^The Pirbright Institute, Pirbright, United Kingdom; ^4^Faculty of Health and Medical Sciences, School of Veterinary Medicine, University of Surrey, Guildford, United Kingdom

**Keywords:** platelet activating factor C-16, PAF analogs, tuberculosis, *Mycobacterium tuberculosis*, *Mycobacterium bovis* BCG, *Mycobacterium smegmatis*, bacterial cell membrane

## Abstract

*Mycobacterium tuberculosis*, the causative agent of tuberculosis, is one of the leading causes of human deaths due to a single infectious agent. *M. tuberculosis* infection of the host initiates a local inflammatory response, resulting in the production of a range of inflammatory factors at the site of infection. These inflammatory factors may come in direct contact with *M. tuberculosis* and immune cells to activate different signaling pathways. One such factor produced in excess during inflammation is a phospholipid compound, Platelet Activating Factor C-16 (PAF C-16). In this study, PAF C-16 was shown to have a direct inhibitory effect on the growth of *Mycobacterium bovis* BCG (*M. bovis* BCG) and *Mycobacterium smegmatis* (*M. smegmatis*) in a dose- and time-dependent manner. Use of a range of PAF C-16 structural analogs, including the precursor form Lyso-PAF, revealed that small modifications in the structure of PAF C-16 did not alter its mycobacterial growth inhibitory properties. Subsequent experiments suggested that the attachment of aliphatic carbon tail via ether bond to the glycerol backbone of PAF C-16 was likely to play a vital role in its growth inhibition ability against mycobacteria. Fluorescence microscopy and flow cytometry using Propidium iodide (PI) indicated that PAF C-16 treatment had a damaging effect on the cell membrane of *M. bovis* BCG and *M. smegmatis*. Furthermore, the growth inhibitory effect of PAF C-16 was partially mitigated by treatment with membrane-stabilizing agents, α-tocopherol and Tween-80, which further suggests that the growth inhibitory effect of PAF C-16 was mediated through bacterial cell membrane damage.

## Introduction

Tuberculosis (TB) is an infectious disease, caused by the acid-fast bacillus, *Mycobacterium tuberculosis*. According to the World Health Organization (WHO), TB is one of the leading causes of human mortality, resulting in more than one million human deaths each year. Approximately 10.4 million new cases of TB were reported worldwide in the year 2016 (World Health Organization [WHO], 2017). It is estimated that about one-third of the world’s population (1.7 billion people) is latently infected with *M. tuberculosis*, a condition with no active disease symptoms due to containment of the pathogen by the host immune system (Houben and Dodd, 2016). This latent infection with *M. tuberculosis* provides a huge reservoir for reactivation into active TB and its spread across the globe. TB continues to be a major health problem due to the non-availability of an effective vaccine. The only preventive vaccine against TB is BCG, which is almost a century old and not effective in controlling the spread of *M. tuberculosis* infections in economically active adult humans, which comprises the major population of TB patients in developing countries (Sepulveda et al., 1992; Colditz et al., 1994; Aronson et al., 2004; Lahey and von Reyn, 2016). New challenges, such as cases of HIV-1 and *M. tuberculosis* co-infection (Daley et al., 1992), multidrug-resistant tuberculosis (MDR-TB) (Espinal et al., 2000), and extensively drug resistant tuberculosis (XDR-TB) (Jain and Mondal, 2008; Liu et al., 2011) have compounded the severity of the problem, and therefore, novel therapeutic interventions are required to control TB.

Infection of a host by *M. tuberculosis* activates the host’s immune system resulting in a localized inflammatory response (Cooper and Flynn, 1995; Giacomini et al., 2001). This causes an increase in blood flow and changes in vascular permeability, resulting in the leakage of several proteinaceous and non-proteinaceous factors and the migration of cellular components from the blood to the site of infection (Sherwood and Toliver-Kinsky, 2004; Clark et al., 2007). In addition to the plasma-derived factors, complement proteins such as C1q and lipid compounds, such as PAF C-16 and Lyso-PAF, are also synthesized by inflammatory cells such as macrophages at the site of infection (Camussi et al., 1987; Kaul and Loos, 1995). These factors are likely to come in contact with the pathogens and immune cells, and thus, may modulate the outcome of the infection.

PAF C-16 or PAF-acether, a membrane-derived phospholipid is chemically known as 1-O-alky-2-acetyl-*sn*-glycero-3-phosphocholine. PAF C-16 is normally present in low amounts in human serum (approximately 127 pg/ml); however, its concentration increases by six-fold during allergic reactions (Vadas et al., 2013). PAF C-16 is produced by a range of cells, including platelets, neutrophils, macrophages, endothelial and mast cells (Alam et al., 1983; Bussolino et al., 1986; Schleimer et al., 1986; Leaver et al., 1990; Biffl et al., 1996; Gardiner et al., 1999). PAF C-16 is synthesized by two distinct pathways; the remodeling pathway and the *de novo* synthesis pathway (Prescott et al., 1990). The remodeling pathway is the major PAF C-16 synthesis pathway used by activated inflammatory cells and involves the modification of membrane ether-linked phospholipids by the enzymes PLA_2_ and acetyl coenzyme A acetyltransferase in a two-step process to produce PAF C-16 (Shindou et al., 2007).

PAF C-16 binds to specific transmembrane G-protein coupled receptors, known as PAF receptor (PAFR), on the plasma membrane of target cells (Honda et al., 2002; Ishii et al., 2002). Binding of PAF C-16 to PAFR results in the activation of different signal transduction mechanisms such as phosphatidylinositol-calcium second messenger system, and the activation of different kinases including protein tyrosine kinase, mitogen-activated protein kinases and protein kinase C pathways (Honda et al., 2002).

PAF C-16 is endowed with diverse biological activities including its well-known ability to cause platelet aggregation (Chignard et al., 1980a,b). PAF C-16 also has important roles in inflammatory and allergic responses (Henderson et al., 2000). PAF C-16 induces apoptosis in neuronal and epidermal cells (Barber et al., 1998; Brewer et al., 2002; Ryan et al., 2008), causes the production of reactive oxygen and nitrogen species by macrophages (Hartung et al., 1983; Rouis et al., 1988; Aliberti et al., 1999; Borges et al., 2017), and plays an important role in angiogenesis (Seo et al., 2004). Dysregulated production of PAF C-16 has been associated with a number of diseases including multiple sclerosis (Hostettler et al., 2002), thrombosis (Mueller et al., 1995), myocardial infarctions (Montrucchio et al., 2000), rheumatoid arthritis (Hilliquin et al., 1995), bronchial asthma (Cuss et al., 1986), acute pancreatitis, and inflammatory bowel disease (Rosam et al., 1986). PAFR antagonists bind to PAFR with high affinity and have been shown to successfully inhibit certain pathological processes in asthma, cardiac, and circulatory disorders that are driven by PAF C-16 (Singh et al., 2013).

There is a limited information available on the direct anti-microbial activity of PAF C-16. PAF C-16 has been shown to inhibit the growth of Gram-positive bacteria in cultures, but not Gram-negative bacteria (Steel et al., 2002). However, the direct anti-mycobacterial role of PAF C-16 has not been investigated. In this study, exogenous PAF C-16 and its structural analogs were examined for their direct effect on mycobacterial growth using *M. bovis* BCG (Pasteur 1173P2) and *M. smegmatis* (mc^2^ 155) as models for *M. tuberculosis*. We report that PAF C-16 and a number of PAF C-16 structural analogs inhibited the growth of both *M. bovis* BCG and *M. smegmatis*. The active portion of PAF C-16 responsible for mycobacterial growth inhibition and the underlying mechanisms of growth inhibition by PAF C-16 were also investigated.

## Materials and Methods

### Chemicals

PAF C-16 (1-O-hexadecyl-2-O-acetyl-*sn*-glyceryl-3-phosphoryl choline), Lyso-PAF C-16 (1-O-hexadecyl-2-hydroxy-*sn*- glyceryl-3-phosphorylcholine), PAF C-18 (1-O-octadecyl- 2-O-acetyl-*sn*-glyceryl-3-phosphorylcholine), Hexanolamino PAF C-16 [1-O-hexadecyl-2-O-acetyl-*sn*-glyceryl-3-phosphoryl (*N*,*N*,*N*-trimethyl) hexanolamine], 2-O-methyl PAF C-16 (1-O-hexadecyl-2-O-methyl-*sn*-glyceryl-3-phosphoryl choline), Pyrrolidino PAF C-16 (1-O-hexadecyl-2-O-acetyl-*sn*-glyceryl-3-phosphoryl-N-methyl-pyrrolidinium ethanol), and Miltefosine (1-hexadecylphosphorylcholine) were obtained from Cayman Chemical Company, United States (**Figure 3**). 1-O-hexadecyl-*sn*-glycerol (Bachem) was obtained from Cambridge Biosciences, United Kingdom, Palmitic acid (Hexadecanoic acid), Vitamin E [(±)-α-Tocopherol] and Phosphocholine chloride calcium salt tetrahydrate were purchased from Sigma-Aldrich Company, United States, and Hexadecyl lactate was purchased from Santa Cruz Biotechnology, United States. All other chemicals used in the experiments were of analytical grade.

Stock solutions of phospholipids and fatty acids were prepared in ethanol (10 mg/ml). Miltefosine was dissolved in PBS (10 mg/ml), Hexadecyl lactate in DMSO (10 mg/ml), and Phosphocholine chloride calcium salt tetrahydrate in water (10 mg/ml). Appropriate solvent controls were included in all the experiments involving PAF C-16, its structural analogs or any other chemical compound used with bacteria.

### Mycobacterial Strains and Growth Conditions

Liquid cultures of *M. smegmatis* (mc^2^ 155) were grown in Luria-Bertani (LB) broth (Lennox; Sigma Aldrich) containing 50 μg/ml carbenicillin (Fisher Chemical), 0.15% (v/v) glycerol and 0.1% (v/v) tween-80 in a shaking incubator at 37°C until the O.D_(600nm)_ reached 0.8–0.9. The number of *M. smegmatis* colony forming units (CFUs) per ml was determined by plating different dilutions of the bacterial stock on LB agar plates in triplicates and counting the number of CFUs after incubation at 37°C for 72 h.

Liquid cultures of *M. bovis* BCG (Pasteur 1173P2) were grown in Middlebrook 7H9 broth (Sigma Aldrich), supplemented with 10% (v/v) albumin dextrose catalase (ADC) and 0.2% (v/v) tween-80. ADC was prepared by dissolving 5 g of bovine albumin fraction V (Fisher Chemical), 2 g dextrose (Fisher Chemical), 0.85 g sodium chloride (Fisher Chemical) and 4 mg catalase from bovine liver (Sigma Aldrich) in 100 ml of sterile water and the solution was passed through a 0.22 μm filter. The growth cultures were kept in a shaking incubator at 37°C for 2–3 weeks until the O.D_(600nm)_ reached 0.8–0.9. The number of CFUs per ml for stock *M. bovis* BCG was determined by plating different dilutions of the bacterial stock on 7H10 plates (BD Biosciences) in triplicates and counting the number of CFUs after incubation at 37°C for 2–3 weeks.

### Mycobacterial Growth Inhibition Assays

Appropriately diluted *M. bovis* BCG and *M. smegmatis* (2.5 × 10^4^) in suspensions of 1ml 7H9 or LB broth, respectively, were exposed to a range of concentrations of PAF C-16 or PAF C-16 analogs (10–100 μg/ml) for 2 h at 37°C with mixing every 15 min. Appropriate solvent controls for the test chemicals were also included in all the experiments. After incubation, 200 μl of bacterial suspensions from test compound treated and solvent control tubes were plated on agar plates in triplicate and viable colony counting methods were used to detect the direct growth inhibitory effects of PAF C-16 and its analogs. *M. smegmatis* was seeded on LB agar plates and incubated for 72 h while 7H10 plates were used for *M. bovis* BCG and incubated for 3–4 weeks at 37°C after which the CFUs were counted by the naked eye.

The above described protocol was also used to investigate *in vitro* direct growth inhibitory effect of Palmitic acid (10–100 μg/ml), Phosphocholine chloride calcium salt tetrahydrate (10–100 μg/ml), 1-O-hexadecyl-*sn*-glycerol (10–100 μg/ml), Miltefosine (10–100 μg/ml), and Hexadecyl lactate (10–100 μg/ml) in order to localize the biologically active portion of PAF C-16 contributing to the growth inhibition.

Additional experiments to determine the effect of incubation time on the growth inhibitory concentration of PAF C-16 against *M. bovis* BCG and *M. smegmatis* were performed in a similar manner. However, lower concentrations of PAF C-16 (1–25 μg/ml) were also used and the incubation periods with bacteria were increased to 6, 12, and 24 h.

### Fluorescence Microscopy and Flow Cytometry to Detect Damage to the Mycobacterial Cell Membrane

Propidium Iodide (PI) (BioLegend, San Diego, CA, United States), a nucleic acid binding fluorescent dye, was used to detect damage to the mycobacterial cell membrane by using fluorescence microscopy and flow cytometry. One ml of *M. bovis* BCG or *M. smegmatis* [O.D_(600nm)_ = 0.9] from fresh culture was washed with PBS via centrifugation (5000 rpm, 10 min), resuspended in 1ml of broth culture media, and then incubated with 100 μg PAF C-16 for 2 h at 37°C with mixing every 15 min. Ethanol treated (10 μl/ml for 2 h) and heat killed (100°C for 10 min) bacterial samples were used as solvent control and positive control, respectively. Next, PAF C-16 treated test and untreated control bacterial samples were washed twice with PBS (5000 rpm for 10 min) and then stained with PI (1 μg/ml) for 20 min at room temperature in dark. The excess dye was removed by washing with PBS.

For fluorescence microscopy, the bacterial pellets were resuspended in 100 μl of PBS, and 5 μl of bacteria from each condition was examined at 400× magnification using a Leica DM4000^®^ fluorescence microscope. For flow cytometry, the bacterial pellets were resuspended in 250 μl of PBS and 25000 events were acquired using ACEA NovoCyte^®^ Flow Cytometer; the acquired data was analyzed by NovoExpress^®^ software. To determine the percentage of PI stained bacteria, first the solvent control (10 μl ethanol/ml of bacterial suspension) stained with PI was analyzed on a density plot by applying gates such that most of the bacterial population was negative for PI. The same gates were then applied on the density plots for heat-killed (100°C for 10 min) positive control and 100 μg/ml PAF C-16 treated test conditions.

### Effect of α-Tocopherol and Tween-80 on PAF C-16 Induced Growth Inhibition

α-tocopherol and tween-80 were used to determine if they can mitigate the PAF C-16 induced bacterial growth inhibition as shown previously (Kagan, 1989; Li et al., 2011). *M. smegmatis* (2.5 × 10^4^) and *M. bovis* BCG (2.5 × 10^4^) were resuspended in 1ml broth medium and treated with either 100 μg/ml α-tocopherol or 1% v/v tween-80 for 1h at 37°C. After 1 h, PAF C-16 (100 μg/ml bacterial suspension) was added to the samples and incubated at 37°C for 2 h with mixing after every 15 min. Solvent (20 μl ethanol/ml bacterial suspension), α-tocopherol (100 μg/ml bacterial suspension), tween-80 (1% v/v bacterial suspension) and PAF C-16 (100 μg/ml bacterial suspension) treated *M. smegmatis* or *M. bovis* BCG were included as controls. After incubation, 200 μl of bacterial suspension from the test and control tubes was seeded on agar plates in triplicate and the CFUs were counted using viable colony counting method for each condition.

### Statistical Analysis

All the experiments were repeated 3–6 times. The data for growth inhibition assays were expressed as mean ± standard error of means (S.E.M), and the solvent treated samples were considered 100% bacterial survival. Statistical analysis was performed using GraphPad Prism^®^ software (Version 5.01) to determine the level of significance (*p*-value) by applying non-parametric multiple comparison Kruskal-Wallis test on ranks and individual data-sets were compared using *post hoc* Dunn’s multiple comparison test. For comparison of two particular datasets, non-parametric Mann-Whitney test was used. A *p*-value of less than or equal to 0.05 (*p* ≤ 0.05) was considered to be significant. On the graphs *p* ≤ 0.05 is denoted by ^∗^, *p* ≤ 0.01 by ^∗∗^ and *p* ≤ 0.001 by ^∗∗∗^.

## Results

### PAF C-16 Inhibits *M. smegmatis* and *M. bovis* BCG Growth in a Dose-Dependent Manner

**FIGURE 1:**
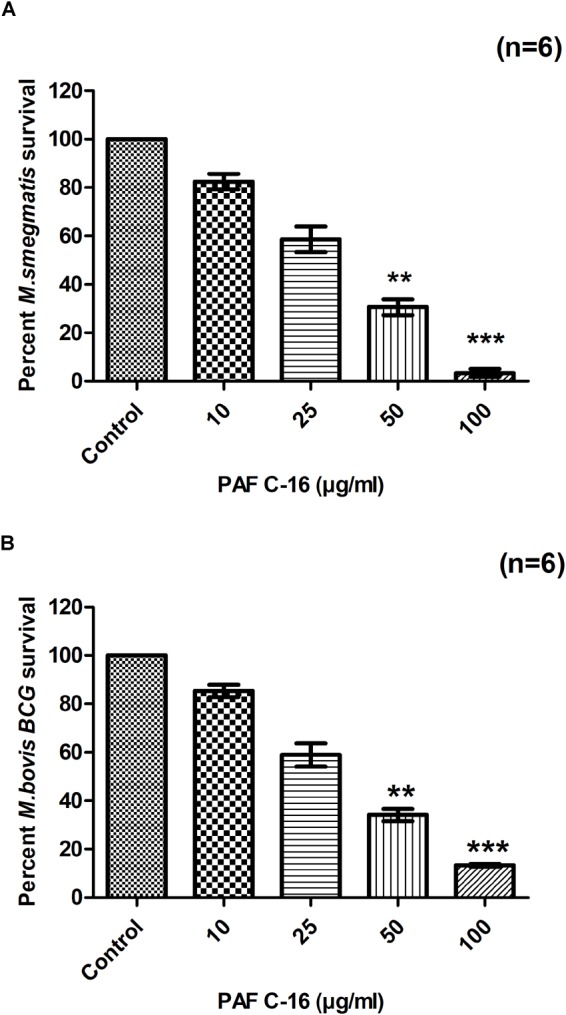
Effect of PAF C-16 treatment on *M. smegmatis* and *M. bovis* BCG survival *in vitro*. Number of surviving colony forming units of *M. smegmatis* **(A)** and *M. bovis* BCG **(B)** after 2 h of treatment with the indicated concentrations of PAF C-16. Data is expressed in terms of percent survival where solvent control is taken as 100% survival and different PAF C-16 treated test conditions are compared to it. Each bar represents the average of six individual experiments performed in triplicate and the error bars show the standard error of mean values (±SEM). Statistically significant differences from the solvent control by Kruskal-Wallis test with *post hoc* Dunn’s multiple comparison test are indicated ^∗∗^*p* ≤ 0.01, ^∗∗∗^*p* ≤ 0.001.

Treatment of *M. smegmatis* and *M. bovis* BCG with PAF C-16 (10, 25, 50, and 100 μg/ml) for 2 h resulted in a dose-dependent growth inhibition, as evident from the decrease in the number of surviving CFUs when compared with PAF C-16 solvent control (10 μl ethanol/ml bacterial culture) (**Figures 1A,B**). PAF C-16 treatment of *M. smegmatis* and *M. bovis* BCG at the lower range of concentrations (10 and 25 μg/ml) showed 15–40% reduction in the number of CFUs. The number of *M. smegmatis* CFUs after treatment with 50 μg/ml PAF C-16 on an average decreased by 65%, whereas, 100 μg/ml PAF C-16 treatment caused 97% reduction (**Figure 1A**). Similar results were obtained for *M. bovis* BCG where 50 μg/ml and 100 μg/ml PAF C-16 treatment on an average reduced the number of surviving CFUs by 66% and 88%, respectively, when compared to the number of CFUs from the solvent control (**Figure 1B**). Although a trend of *M. smegmatis* and *M. bovis* BCG growth inhibition was seen in all the experiments using the above range of PAF C-16 concentrations, only PAF C-16 at the concentration of 50 μg/ml (*p* ≤ 0.01) and 100 μg/ml (*p* ≤ 0.001) reached statistical significance using stringent non-parametric Dunn’s multiple comparison test.

### PAF C-16 Inhibits *M. smegmatis* and *M. bovis* BCG Growth in a Time-Dependent Manner

**FIGURE 2:**
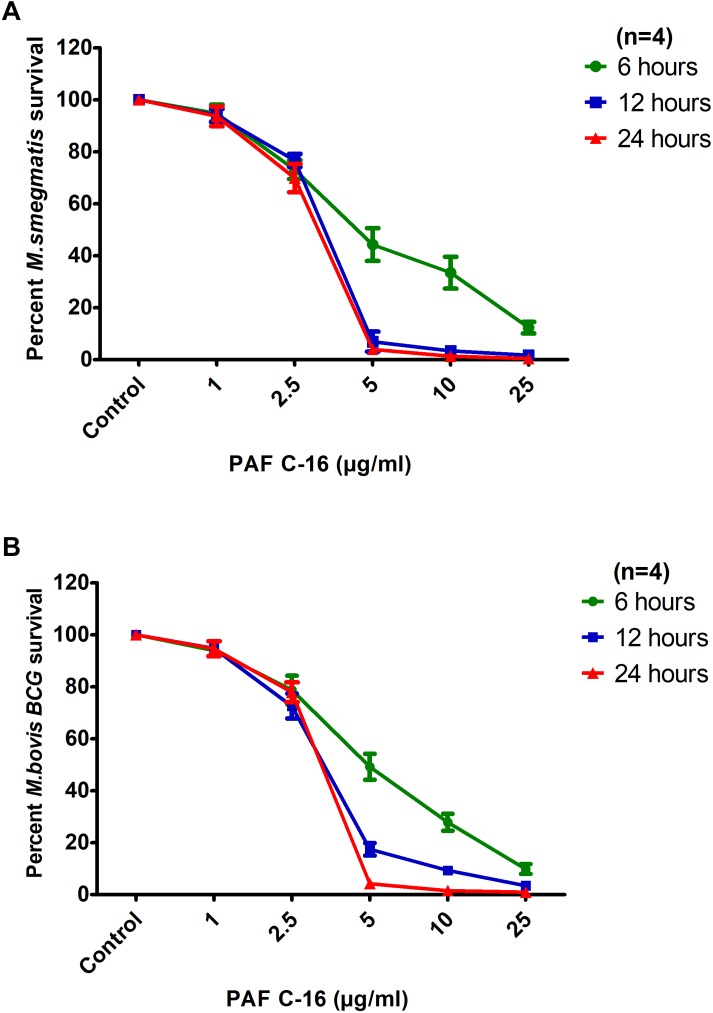
Inhibitory effects of PAF C-16 on *M. smegmatis* and *M. bovis* BCG growth at increased incubation durations. Number of surviving colony forming units of *M. smegmatis* **(A)** and *M. bovis* BCG **(B)** after 6, 12, and 24 h of treatment with indicated concentrations of PAF C-16. Data is expressed in terms of percent survival where solvent control is taken as 100% survival. Each data point represents the average of four individual experiments performed in triplicate and the error bars show the standard error of mean values (±SEM).

To assess the effect of PAF C-16 exposure time on the growth inhibition of *M. smegmatis* and *M. bovis* BCG, bacteria were treated for extended durations of 6, 12 and 24 h with PAF C-16 (1–25 μg/ml) (**Figures 2A,B**). It was observed that in addition to concentration, the growth inhibition of both *M. smegmatis* and *M. bovis* BCG by PAF C-16 was also dependent on the treatment duration. PAF C-16 treatment at 5 μg/ml or at higher concentrations was effective in inhibiting the growth of both *M. smegmatis* and *M. bovis* BCG by ≥50% at all the three time points. At 6 h, PAF C-16 treatment at a concentration of 5 μg/ml caused a reduction of 55% in the number *M. smegmatis* CFUs when compared to the solvent control. The PAF C-16 caused *M. smegmatis* growth inhibition at 5 μg/ml increased to ≥95%, as evident from the decrease in CFUs when the treatment times were increased to 12 and 24 h (**Figure 2A**). Similar results were obtained with *M. bovis* BCG. At 6 h, PAF C-16 at the concentration of 5 μg/ml reduced the number of *M. bovis* BCG CFUs by 51% which increased to 85% after 12 h treatment and finally reached 96% after 24 h (**Figure 2B**). PAF C-16 at the concentration of 2.5 μg/ml after 6 h treatment on an average caused 14% and 12% reduction in the number of *M. smegmatis* and *M. bovis* BCG CFUs, respectively, which increased to ∼29% after 24 h treatment. Furthermore, 1 μg/ml PAF C-16 showed negligible growth inhibition at all the three time points tested against both *M. smegmatis* and *M. bovis* BCG.

Subsequent experiments with PAF C-16 and its structural analogs were performed at concentrations ranging from 10 to 100 μg/ml with treatment duration of 2 h, because we established the growth inhibition of *M. smegmatis* and *M. bovis* BCG at the lowest concentration of 5 μg/ml PAF C-16 at increased treatment durations.

### PAF C-16 Analogs Show a Similar Growth Inhibitory Effect Against *M. smegmatis* and *M. bovis* BCG

**FIGURE 3:**
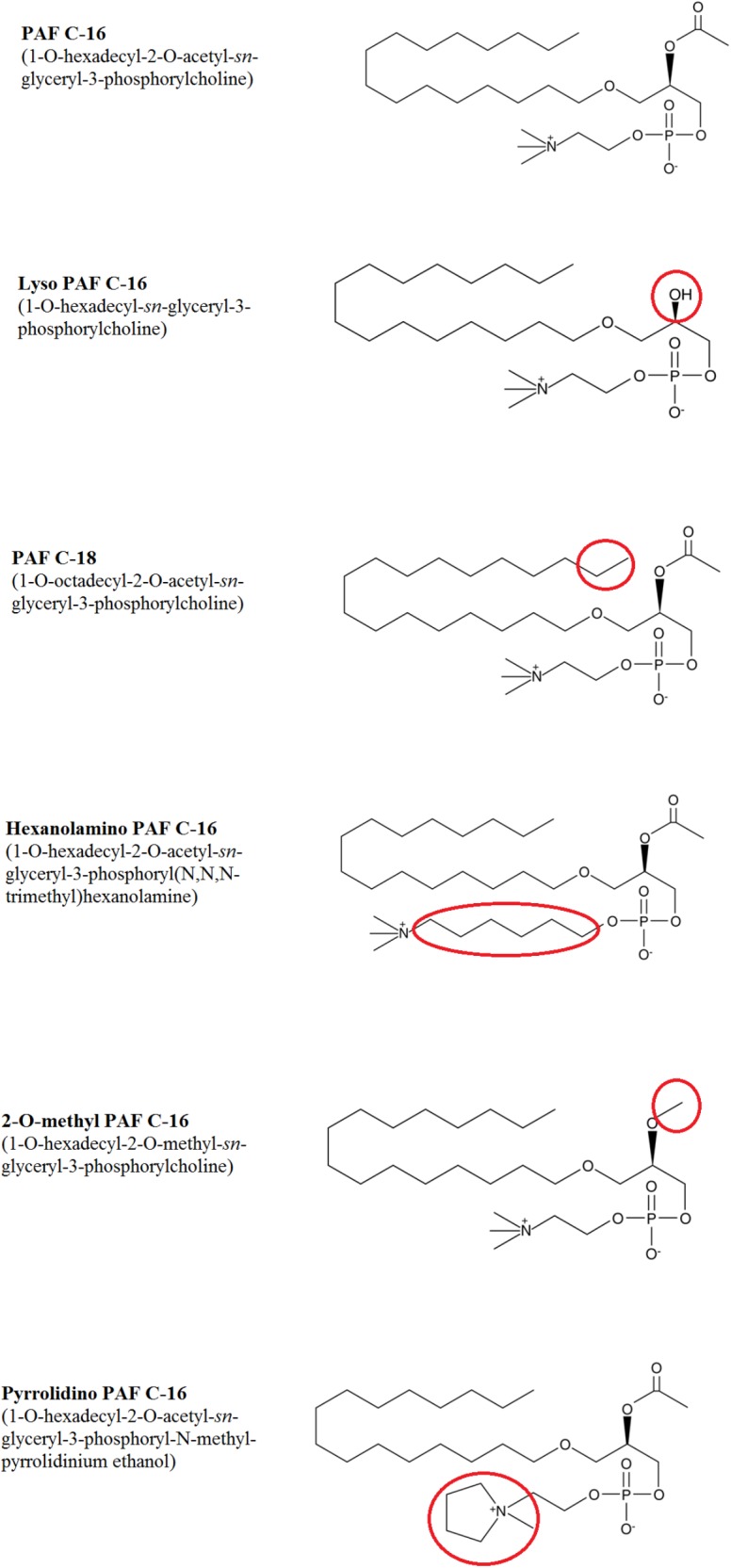
Chemical structures of PAF C-16 and its structural analogs. Each analog has a small modification in the structure as compared to naturally occurring PAF C-16 which is highlighted in the red circles.

**FIGURE 4:**
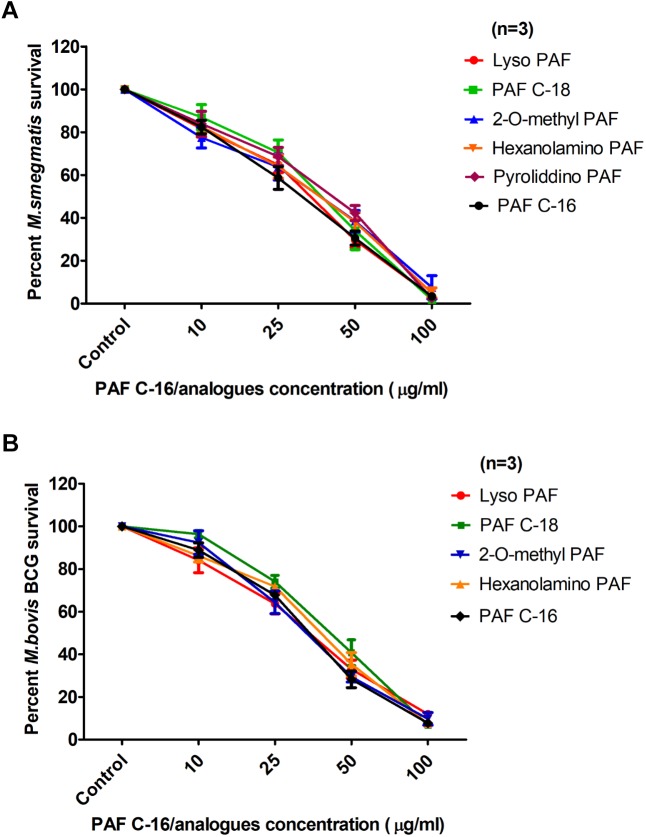
Comparison of the levels of growth inhibition by different PAF C-16 analogs and PAF C-16 for *M. smegmatis* and *M. bovis* BCG. Number of surviving colony forming units of *M. smegmatis* **(A)** and *M. bovis* BCG **(B)** after 2 h treatment with indicated concentrations of different PAF C-16 analogs and PAF C-16. The data is expressed as percentage survival where solvent control is taken as 100% survival. Each data point represents the average of three individual experiments performed in triplicate and the error bars show the standard error of mean values (±SEM).

Different PAF C-16 structural analogs, including the naturally occurring precursor form Lyso-PAF C-16, PAF C-18 and Hexanolamino PAF C-16 as well as synthetic analogs, such as 2-O-methyl PAF C-16 and Pyrrolidino PAF C-16 were tested against both *M. smegmatis* and *M. bovis* BCG in order to assess the impact of small modifications of the structure of PAF C-16 on the *in vitro* bacterial growth inhibition potential (**Figure 3**). PAF C-16 structural analogs used were selected such that each analog represented a change in different functional groups when compared to PAF C-16. These PAF C-16 structural analogs were able to inhibit the growth of both *M. smegmatis* and *M. bovis* BCG in a dose-dependent manner, when tested at the concentrations ranging between 10 and 100 μg/ml (**Figures 4A,B**). *M. smegmatis* and *M. bovis* BCG growth inhibition (*p* ≤ 0.01–*p* ≤ 0.001) was significant at 50 μg/ml (≤50% reduction in CFUs) and 100 μg/ml (≤90% reduction in CFUs) for all PAF C-16 analogs tested. Furthermore, the mycobacterial growth inhibition potency of these PAF C-16 structural analogs was comparable to PAF C-16. This showed that small alterations in the structure of PAF C-16 did not affect its direct growth inhibitory potential against mycobacteria.

### Structural Dissection of PAF C-16 to Localize the Anti-microbial Active Portion

**FIGURE 5:**
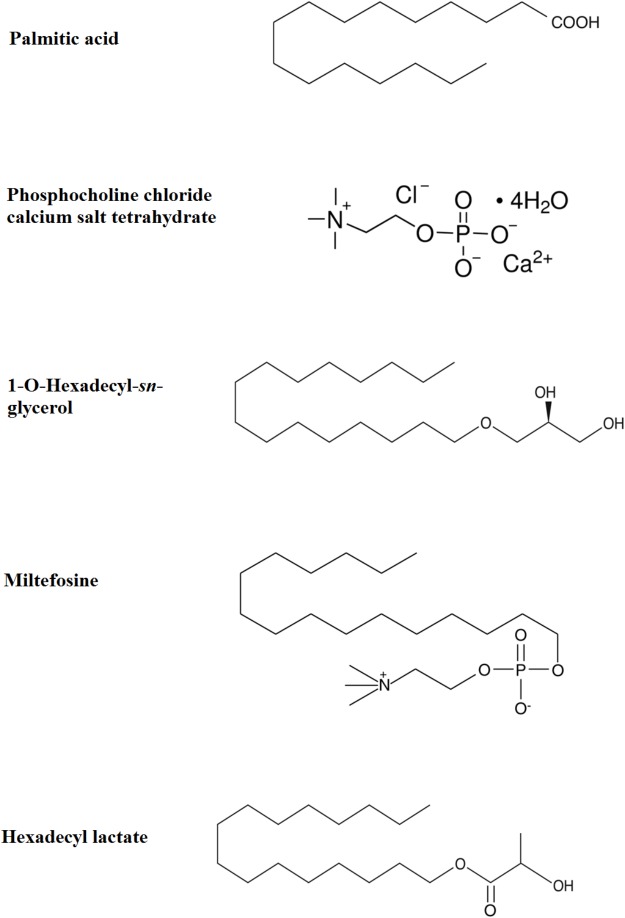
Chemical structures of Palmitic acid, Phosphocholine chloride calcium tetrahydrate, 1-O-Hexadecyl-*sn*-glycerol, Miltefosine, and Hexadecyl lactate.

**FIGURE 6:**
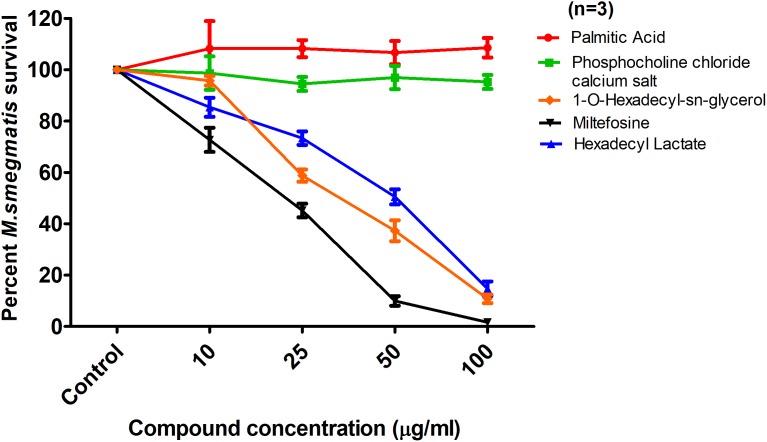
Effect of Palmitic acid, Phosphocholine chloride calcium tetrahydrate, 1-O-hexadecyl-*sn*-glycerol, Miltefosine, and Hexadecyl lactate on *M. smegmatis* survival. The data is expressed as percentage where solvent control is taken as 100% survival. Each data point represents the average of three individual experiments performed in triplicate and the error bars show the standard error of mean values (±SEM).

To localize the biologically active portion of PAF C-16 that contributed to its growth inhibitory properties against mycobacteria, compounds with structures similar to different portions of PAF C-16 (**Figure 5**) were investigated for their direct mycobacterial growth inhibitory potential using *M. smegmatis* as a model. Palmitic acid (hexadecanoic acid) having a linear 16-carbon atoms chain similar to the carbon tail of PAF C-16 in the number of carbon atoms, was tested to determine the role of the carbon chain in the growth inhibition activity of PAF C-16. However, Palmitic acid (10–100 μg/ml) did not show any inhibitory effect on *M. smegmatis* growth; in fact, it showed a slight growth enhancing effect (**Figure 6**). Phosphocholine chloride calcium tetrahydrate salt with a structure resembling the phosphocholine head region of PAF C-16 also did not show any direct inhibitory effect on the growth of *M. smegmatis* (**Figure 6**). Finally, 1-O-hexadecyl *sn*-glycerol, a compound with a 16-carbon atoms tail attached via ether bond to a glycerol backbone as in PAF C-16, showed direct dose-dependent growth inhibition of *M. smegmatis* following 2 h treatment (**Figure 6**). This suggested that the attachment of a carbon tail via an ether linkage may be important for the anti-mycobacterial characteristics of PAF C-16. Further experiments with other compounds, such as miltefosine and hexadecyl lactate, each having a 16-carbon atoms tail attached via ester linkage to a phosphate and lactyl group, respectively, also revealed growth inhibition of *M. smegmatis* (**Figure 6**).

### PAF C-16 Causes Damage to the Mycobacterial Cell Membrane

Fluorescence microscopy and flow cytometry were performed to assess the damaging effect of PAF C-16 on the cell membrane integrity of mycobacteria. The cell membrane integrity was determined by using a nucleic acid binding fluorescent dye, Propidium Iodide (PI). PI only enters the bacteria when the cell membrane is damaged while bacteria with intact cell membranes are impermeable to this dye (Cox et al., 2000).

**FIGURE 7:**
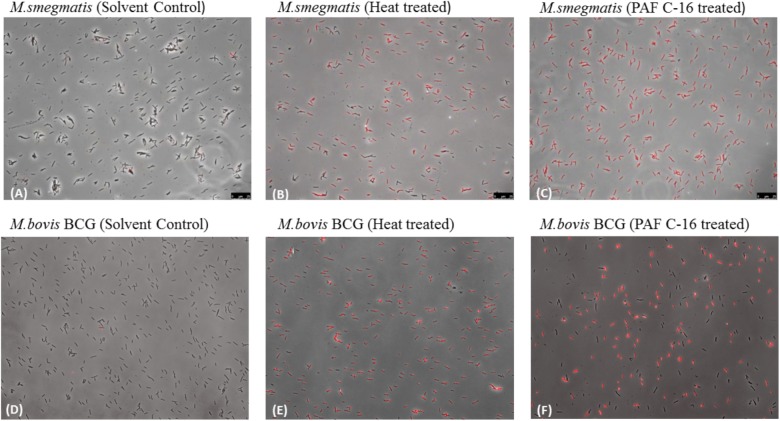
Fluorescence microscopy to detect the effect of PAF C-16 treatment on *M. smegmatis* and *M. bovis* BCG cell membrane integrity. *M. smegmatis* **(A–C)** and *M. bovis* BCG **(D–F)** treated with PAF C-16 (100 μg/ml) or solvent control (10 μl/ml ethanol) for 2 h and heat killed (100°C, 10 min) as a positive control were stained with the nucleic acid binding dye propidium iodide and imaged at 400× magnification in bright field and CY3 (PI) channels. Panels show a merger of the two channels. **(A,D)** are solvent treated negative control, **(B,E)** are heat treated positive control, **(C,F)** are PAF C-16 treated test.

The qualitative analysis of *M. smegmatis* and *M. bovis* BCG by fluorescence microscopy following treatment with 100 μg/ml PAF C-16 for 2 h revealed loss of membrane integrity, as determined by positive staining with PI (**Figures 7C,F**). No PI staining was observed in the solvent control (10 μl ethanol/ml of bacterial suspension), suggesting that the bacterial membrane was intact (**Figures 7A,D**). Heat-killed (100°C for 10 min) *M. smegmatis* and *M. bovis* BCG were also included as a positive control and majority of the bacteria stained positively with PI (**Figures 7B,E**). These results from fluorescence microscopy suggested that PAF C-16 had a damaging effect on the cell membranes of both *M. smegmatis* and *M. bovis* BCG that can lead to the growth inhibition of these bacteria.

**FIGURE 8:**
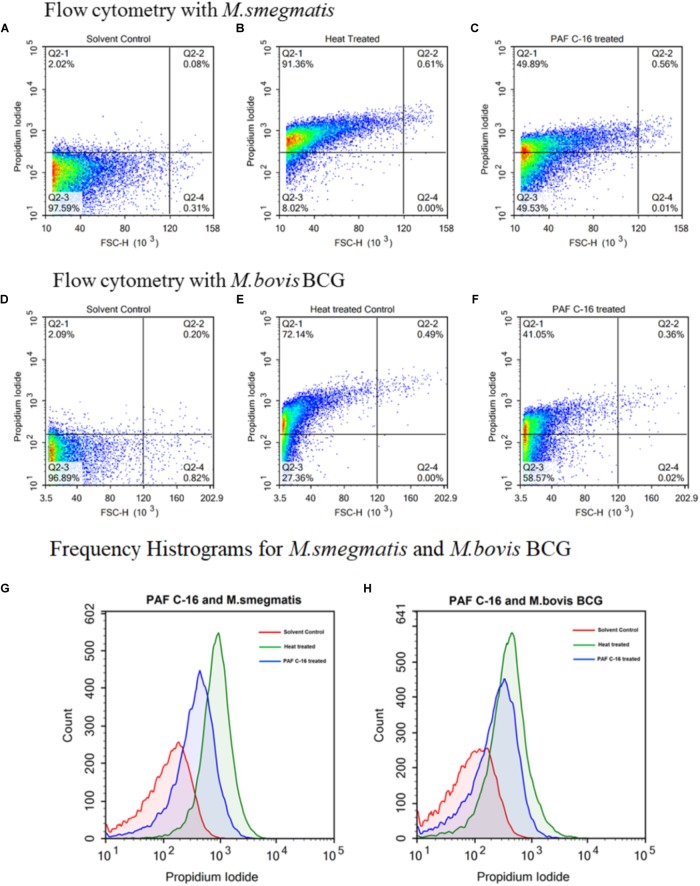
Flow cytometric analysis to determine cell membrane integrity of PAF C-16 treated *M. smegmatis* and *M. bovis* BCG. Flow cytometry density plots for *M. smegmatis* **(A–C)** and *M. bovis* BCG **(D–F)** treated with PAF C-16 or solvent control for 2 h and heat killed positive control after staining with propidium iodide. Density plots **(A,D)** are solvent treated (10 μl/ml ethanol) negative control, density plots **(B,E)** are heat treated (100°C, 10 min) positive control, density plots **(C,F)** are PAF C-16 treated (100 μg/ml) test conditions. Each plot shows percentage of unstained and PI stained bacteria for different conditions. The difference in fluorescence intensity for PAF C-16 treated test, solvent treated negative control and heat treated positive control are shown using frequency histogram overlay for *M. smegmatis* **(G)** and *M. bovis* BCG **(H)**. The blue color histograms represent PAF C-16 treated bacteria, red color is for solvent treated negative control and green color represents heat treated positive control.

To further quantify the percentage of injured/dead bacteria, flow cytometry was performed using *M.* *smegmatis* and *M. bovis* BCG treated with 100 μg/ml PAF C-16 for 2 h. It was observed that ∼50% of *M. smegmatis* were stained with PI following treatment with PAF C-16, whereas, only 2% of *M. smegmatis* were stained with PI in the solvent control and 91% staining was observed for the positive control heat-killed *M. smegmatis* (**Figures 8A–C**). Similar results were obtained with *M. bovis* BCG; 100 μg/ml PAF C-16 treatment resulted in ∼41% of bacteria being stained with PI as compared to 2% PI positive staining in solvent control and 72% staining in heat-killed *M. bovis* BCG (**Figures 8D–F**). The histogram overlays (**Figures 8G,H**) showed a more than four-fold increase in the mean channel fluorescence (MeanX) for PAF C-16 treated *M. smegmatis* (MeanX = 318) and *M. bovis* BCG (MeanX = 135) when compared to the solvent only controls for *M. smegmatis* (MeanX = 74) and *M. bovis* BCG (MeanX = 27), respectively. These flow cytometry results reaffirmed the notion that PAF C-16 induced growth inhibition in *M. smegmatis* and *M. bovis* BCG was through damage to their cell membrane.

### α-Tocopherol and Tween-80 Prevent the Growth Inhibitory Effect of PAF C-16 on Mycobacteria

**FIGURE 9:**
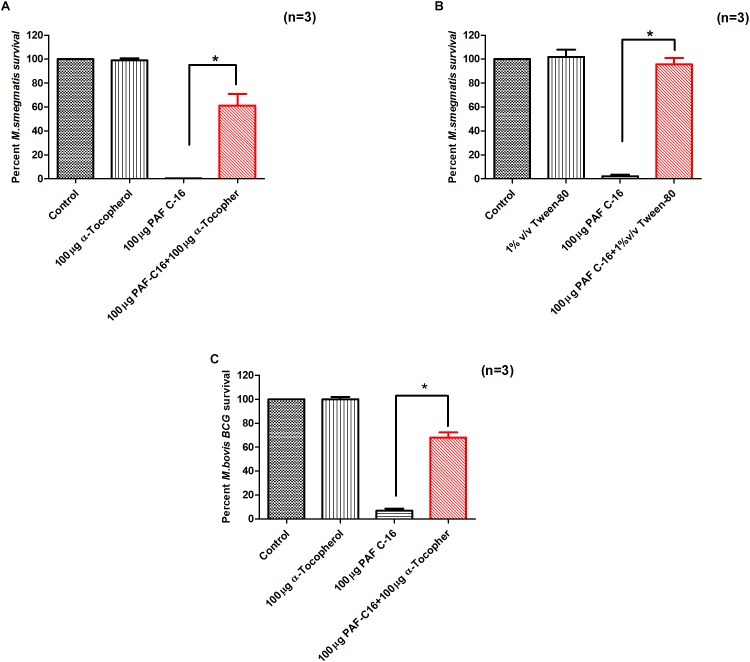
α-Tocopherol and Tween-80 partially mitigate the growth inhibitory effect of PAF C-16 against *M. smegmatis* and *M. bovis* BCG. Number of surviving colony forming units of *M. smegmatis* **(A,B)** and *M. bovis* BCG **(C)** after treatment with the indicated concentrations of α-tocopherol/tween-80 and PAF C-16. The data is expressed as percentage survival where solvent control is taken as 100% survival. Each bar represents the average of three individual experiments performed in triplicate and the error bars show the standard error of mean values (±SEM). The *p*-value was calculated by applying Mann-Whitney test on the given two data sets and was found to be significant ^∗^(*p* ≤ 0.05).

Since PAF C-16 showed damaging effects on both *M.* *smegmatis* and *M. bovis* BCG cell membranes, compounds such as α-tocopherol and tween-80 were used to assess whether they can mitigate the growth inhibitory effect of PAF C-16, and thus, have protective effects on mycobacteria. Both α-tocopherol and tween-80 have previously been shown to reduce the anti-bacterial activity of hydrophobic compounds such as fatty acids, phospholipids and antibiotics like rifampicin (Kagan, 1989; Li et al., 2011; Nielsen et al., 2016). The addition of either α-tocopherol (100 μg/ml) or tween-80 (1% v/v) to *M. smegmatis* or *M. bovis* BCG suspension for 1 h prior to PAF C-16 (100 μg/ml) treatment partially mitigated the inhibitory effect of PAF C-16 on the growth of bacteria as indicated by the increase in number of surviving CFUs on the agar plates when compared with the number of CFUs from bacteria treated with 100 μg/ml PAF C-16 only (**Figures 9A–C**). Furthermore, both α-tocopherol and tween-80 did not show any direct effect on the growth of either *M. smegmatis* or *M. bovis* BCG on their own.

## Discussion

PAF C-16 belongs to a class of single-chained ether linked lipids and is produced by a variety of immune cells including monocytes, macrophages (Elstad et al., 1988) and neutrophils (Lynch et al., 1979). The production of PAF C-16 is enhanced several fold at the site of infection, where it is available to interact with invading pathogens (Huseyinov et al., 1999). However, there is limited information regarding the direct effects of this pro-inflammatory phospholipid on the growth of bacterial pathogens both *in vivo* and *in vitro*. A previous study showed that PAF C-16 can directly inhibit the growth of Gram-positive bacteria (*Staphylococcus aureus and Staphylococcus epidermidis*) *in vitro*, without affecting the growth of Gram-negative bacteria (*Escherichia coli* and *Pseudomonas aeruginosa*) (Steel et al., 2002). In the current study, we have shown, for the first time, that PAF C-16 and its structural analogs can directly inhibit the growth of mycobacteria using *M. smegmatis* and *M. bovis* BCG as model organisms, in a dose- and time-dependent manner *in vitro*.

We first investigated the effect of PAF C-16 on the growth of *M. smegmatis* and *M. bovis* BCG at various concentrations (10–100 μg/ml) and for a shorter treatment duration (2 h), which revealed a decrease in the surviving bacterial CFUs in a dose-dependent manner, suggesting that PAF C-16 had a direct growth inhibitory effect *in vitro*. Since PAF C-16 at higher concentrations can have certain adverse side effects on the host (Stafforini et al., 2003), we further investigated the effect of PAF C-16 on *M. smegmatis* and *M. bovis* BCG growth at lower concentrations (1–25 μg/ml) and at increased treatment durations of 6, 12 and 24 h. We observed that this compound was effective at a concentration as low as 5 μg/ml after 6 h of treatment. In order to get an insight into the structure-function relationships of PAF C-16, different structural analogs were assessed *in vitro* for their direct growth inhibitory potential against *M. smegmatis* and *M. bovis* BCG. PAF C-16 analogs have minor but subtle structural modifications such as variations in functional groups or carbon tail length (Tence et al., 1981; Prescott et al., 1990). Previous studies with PAF C-16 analogs in mammalian systems have shown that small structural modifications can alter the biological activity of PAF C-16 to varying degrees (Shigenobu et al., 1985; O’Flaherty et al., 1987; Rose et al., 1990; Stewart and Grigoriadis, 1991). These PAF C-16 analogs have not been investigated for their direct effect on the growth of mycobacteria.

Lyso-PAF C-16, the precursor form of naturally produced PAF C-16, has a hydroxyl group at position *sn*-2 instead of an acetyl group and is considered to be biologically inactive (Pendino et al., 1993; Aliberti et al., 1999; Montrucchio et al., 2000). In our experiments, however, Lyso-PAF was effective in inhibiting the growth of both *M. smegmatis* and *M. bovis* BCG at the level similar to PAF C-16. Since Lyso-PAF is the inactive precursor form and does not possess most of the side effects associated with PAF C-16, this compound may have therapeutic potential and needs further investigation for its anti-*M. tuberculosis* properties. Similar mycobacterial growth inhibition results were obtained with other PAF C-16 structural analogs including PAF C-18 (18 carbon atoms chain at *sn-1*), Hexanolamino PAF C-16 (additional 4-carbon atoms chain attached to terminal amino group), 2-O-methyl PAF (methyl group at *sn*-2) and Pyrrolidino PAF (5-member lactam ring attached to the phosphate group), indicating that these small modifications had insignificant effects on the bacterial growth inhibition potential.

Structurally, PAF C-16 is composed of a glycerol backbone with a single chained aliphatic carbon tail attached via ether bond at position *sn*-1, an acetyl group at position *sn*-2 and a phosphocholine group attached at *sn*-3 position (Prescott et al., 1990; Venable et al., 1993). Different compounds with structures similar to the phosphocholine head and the aliphatic carbon tail of PAF C-16 were tested to identify the structurally active portion of PAF C-16, which might be involved in the mycobacterial growth inhibition using *M. smegmatis* as a model. It seems that the aliphatic carbon chain linked via oxygen bond to the rest of the molecule is essential for the anti-mycobacterial activity. This is supported by our results which showed that compounds containing such a structure (1-O-hexadecyl-*sn*-glycerol, miltefosine, and hexadecyl lactate) possessed anti-mycobacterial activity, whereas palmitic acid although containing aliphatic carbon chain, but lacking oxygen bond, showed no such growth inhibitory activity. To the best of our knowledge, there is currently no information about the inhibitory effect of these compounds on mycobacterial growth and our results show a novel activity for these compounds. Miltefosine has previously been shown to kill *Leishmania* (Jha et al., 1999; Machado et al., 2010), pathogenic bacteria, such as *Streptococcus pneumoniae* (Llull et al., 2007) and fungi, such as *Aspergillus fumigatus* and *Candida* species (Widmer et al., 2006; Biswas et al., 2013). Similarly, hexadecyl lactate has also been shown to be safe in humans and is used as an additive in food, medicines, and personal care products (Clary et al., 1998; Zhang et al., 2010).

Most of the biological activities of PAF C-16 in mammalian systems are carried out by its binding to specific G-protein coupled receptors, known as PAFR (Honda et al., 2002; Ishii et al., 2002). Currently, it is not known if any PAF C-16 receptor exists on bacteria. However, certain activities attributed to PAF C-16 are independent of its receptor binding, such as its incorporation into biological membranes, which can affect the molecular organization of membrane lipids, and hence membrane functions (Sawyer and Andersen, 1989). In addition, very limited research has been done to establish the mechanism through which PAF C-16 can inhibit the growth of prokaryotic organisms. Exogenous PAF C-16 affects the cell membrane in Gram-positive bacteria by causing the dysfunction of potassium ion (K^+^) transport and leads to the bacterial death but it is ineffective against Gram-negative bacteria (Steel et al., 2002). In our study, fluorescence microscopy and flow cytometry analysis to determine the effect of PAF C-16 on the cell membrane of *M. smegmatis* and *M. bovis* BCG revealed positive PI staining, which occurred due to the entry of the dye in these bacteria, indicating damage to the bacterial cell membrane. Furthermore, prior treatment of the bacteria with α-tocopherol, which is a well-known anti-oxidant and membrane stabilizer (Kagan, 1989; Urano et al., 1992), partially mitigated the PAF C-16 induced bacterial growth inhibition. However, ascorbic acid, another compound with anti-oxidant properties, failed to prevent PAF C-16 induced growth inhibition of *M. smegmatis* (data not included), suggesting that the protective role of α-tocopherol is through its membrane stabilizing mechanism. Similarly, another membrane stabilizing agent tween-80 (Li et al., 2011), also mitigated the inhibitory effect of PAF C-16 on the growth of *M. smegmatis*. This hypothesis is further supported by a previous study in which tween-80 was shown to reduce the activity of anti-microbial compounds such as essential oils, hydrophobic antibiotics like rifampicin and bile salts (Li et al., 2011; Nielsen et al., 2016). Thus, it is evident that PAF C-16 mediated growth inhibition of *M. smegmatis* and *M. bovis* BCG is via cell membrane damage, as membrane stabilizing compounds successfully prevented the PAF C-16 induced growth inhibition of mycobacteria. However, these membrane stabilizing compounds also have the ability to form micelles in aqueous solutions due to their amphipathic nature (Aizawa, 2009) and there is a possibility that the increased survival of mycobacteria when treated with both α-tocopherol/tween-80 and PAF C-16 might be due to the sequestration of PAF C-16 in these micelles.

In summary, PAF C-16 inhibits the growth of both *M. smegmatis* and *M. bovis* BCG in a dose- and time-dependent manner. The growth inhibitory effect of PAF C-16 seems to be through damage to the mycobacterial cell membrane. The presence of an ether bond at position *sn*-1 along with a 16 atom carbon chain seems to be important in conferring the bacterial growth inhibition potential to PAF C-16. Small structural modifications of PAF C-16 do not affect growth inhibitory potential against these mycobacteria. Since these PAF C-16 analogs may lack most of the biological side effects associated with PAF C-16, they have the potential to be used as anti-TB drugs. Miltefosine, a compound structurally related to PAF C-16 investigated in this study, is currently used for treating Leishmaniasis in humans and has been shown to be well tolerated at 100 mg orally per day (Sundar et al., 2002; Soto et al., 2004). Furthermore, these compounds can be delivered via inhaler at a higher dose directly to the disease site as in the case of pulmonary TB. In this respect, one of the anti-TB drugs, capreomycin, has already been tested in a phase I clinical trial in healthy subjects. In this single-dose escalation study, the maximum dose delivered was 300 mg by handheld inhaler into the lung with minimum side effects (Dharmadhikari et al., 2013). It is worthwhile to note that differences exist between *M. tuberculosis* and *M. bovis* BCG (as well as *M. smegmatis*) with respect to the composition of the cell wall components. Therefore, these PAF C-16 structurally related compounds need further investigation both *in vitro* and *in vivo* using *M. tuberculosis*.

## Author Contributions

AP and MR conceived and designed the study. This work was supervised by AP. MR wrote the manuscript with contributions from AP, UK, and SB. MR carried out majority of the experiments with contributions from AK and SS. MR, AP, and AK analyzed the data. All the authors reviewed the final version of the manuscript approved for submission.

## Conflict of Interest Statement

The authors declare that the research was conducted in the absence of any commercial or financial relationships that could be construed as a potential conflict of interest.
